# Mitigation of pacing-induced cardiomyopathy with superficial right ventricular midseptal pacing: Failure of stylet-driven leads to duplicate 3830 success

**DOI:** 10.1016/j.hroo.2025.05.031

**Published:** 2025-06-06

**Authors:** Kenneth Wade McBride, Nitin K. Kulkarni, Charles C. Hickman, Mark S. Link, Theodore S. Takata

**Affiliations:** 1 Texas Health Heart and Vascular Specialists, Fort Worth, Texas; 2 Department of Cardiology, UT Southwestern Medical Center, Dallas, Texas

**Keywords:** Pacing-induced cardiomyopathy, Right ventricular midseptal pacing, Cardiomyopathy, Pacing, Pacemaker complications

**Key Findings**

▪ Traditional methods of right ventricular (RV) pacing have resulted in significant occurrences of pacing-induced cardiomyopathy.
▪ Active fixation 3830 leads placed in the superficial RV midseptum using C315HIS delivery catheters have recently been demonstrated to mitigate the occurrence of pacing-induced cardiomyopathy.
▪ Our experience with stylet-driven leads did not achieve the same benefit.

Pacing-induced cardiomyopathy (PICM) is an established potential complication of chronic right ventricular (RV) pacing therapy. We have recently demonstrated a significant mitigation of this complication by superficial RV midseptal (RVMS) pacing in patients with normal left ventricular (LV) function vs standard RV apical pacing.^1^ The designation of superficial is used to differentiate this approach from the deep septal approach used for left bundle branch area pacing. These data represented the first to demonstrate a clear improvement from apical pacing, despite attempts of multiple previous studies over 20 years.^2^ We proposed that one potential explanation for this success was the use of septal-specific guide catheters to place the RVMS lead. Most previous studies used stylet-driven leads, or first-generation single-plane deflectable guide catheters. To further explore this potential benefit of septal-specific guide catheters, we examined our experience with stylet-driven leads used for RVMS pacing compared with the Medtronic 3830 lead.

This was a retrospective study of all pacemaker implantations performed at 2 centers in Fort Worth, TX, by 2 electrophysiologists (K.W.M. and N.K.K.) between 2011 and 2022. The study adhered to the Declaration of Helsinki guidelines for human research and was approved by the University of Texas Southwestern Institutional Review Board and the Texas Health Resources Human Research Protection Program. The primary comparison was the relative occurrence of PICM between 2 patient groups with attempted superficial RVMS lead placement: those with Medtronic 3830 or 5076/5086 (stylet-driven) leads. During this time frame, the preferred lead placement technique was the 3830 placed with the septal-specific C315HIS guide catheter. However, owing to the recurring unavailability of the 3830 lead, other techniques were used, including superficial RVMS placement of a stylet-driven lead. PICM was defined as a decline in the LV ejection fraction (LVEF) of at least 10%, with a resulting LVEF of less than 50%. The LVEF measurements were from clinically indicated echocardiograms or other LV imaging techniques.

Patients included in the study were those with normal baseline LVEF, ≥ 20% RV pacing burden, and available baseline and follow-up (F/U) measurements of LVEF. The F/U LVEF measurements had to be at least 6 months after implantation and were interpreted independently of this study. Patients were excluded for biventricular devices, baseline LVEF < 50%, atypical lead location on reviewed chest radiographs, or an alternative cause of cardiomyopathy, as previously reported.^1^

For superficial RVMS placement with a Medtronic model 3830 lead, a nondeflectable Medtronic C315HIS guide catheter was used, as previously described.^1^ The lead was fixated to the superficial midseptal endocardium with just 4 rotations, not intending to “drive” the lead into the deep septum. When the 3830 lead was not available, at times traditional stylet-driven leads were attempted to be placed at the RVMS, without guiding catheters. The stylet was preformed by the implanter, with a U shape placed in the terminal 10–15 cm, with a diameter of approximately 5 cm, and a second curve to the left in the terminal straight portion. Mean, median, minimum, and maximum were calculated for continuous variables, with means compared with a 2-sample *t* test. Numbers and percentages are reported for categorical variables and compared with a χ^2^ test of independence. All analysis was performed using R Statistical Software (version 4.2.1; R Core Team 2022).

Of all patients paced from January 2011 to 2021, 224 with 3830 RVMS pacing and 48 with stylet-driven RVMS pacing met the above criteria. Baseline characteristics for the 2 groups had no statistically significant differences. The median percentage of RV pacing throughout the F/U period was ≥ 99% for both groups.

**Figure 1:**
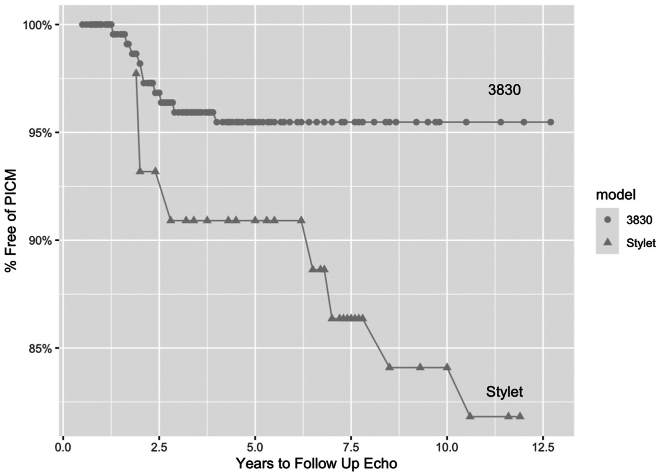
Survival free from PICM, tracked by time to diagnosis, that is, years to follow-up echocardiogram, 3830 lead cohort vs stylet-driven lead cohort (*P* = .00587). Echo = echocardiogram; PICM = pacing-induced cardiomyopathy.

The primary result found is the significantly lower incidence of PICM in the patient population with RVMS pacing performed with the 3830 and septal guide catheters than with stylet-driven leads without guides (Figure 1). Only 4.5% of RVMS paced patients developed PICM vs 16.7% in the stylet-driven leads (*P* = .00587), a 3.7-fold reduction in incidence. Paced QRS duration was also significantly narrower with the 3830 patients, with a mean of 144 ± 15.7 ms, than stylet-driven lead paced patients with a mean of 158 ± 14.6 ms (*P* < .001).

These data indicate that the lead implantation technique used, specifically the utilization of a septal-specific guide catheter to optimize true septal lead placement, is an important determinant of long-term clinical success for RVMS pacing. Although the study is retrospective and not randomized, patient selection was made by lead availability, not physician decision. Another limitation is the less standardized technique for stylet-driven leads, which likely resulted in attachment in less desirable areas of the septum or the free wall or anterior RV/LV trough, compared with the 3830 leads. However, that is the main emphasis of the report: the C315HIS catheter achieves better and more consistently optimal septal attachment. This delivery system and technique has been independently evaluated in a study with postimplant computed tomography imaging, and a high level of accurate septal placement was documented.^3^ Most previous studies looking at the potential benefit of septal pacing used classical stylet-driven active fixation leads, perhaps contributing to their failure to demonstrate any improvement over RV apical pacing. One study clearly indicated that frequent inadvertent RV anterior free wall placement by a standard stylet-driven lead (almost 50% in their study) led to reduced LV function over time, but not in patients with a true midseptal lead.^4^ We submit that, for RVMS pacing, the utilization of septal-specific lead delivery catheters should be considered a preferred lead placement technique, resulting in extremely good clinical outcomes over more than 15 years.
